# A Pre-Clinical Large Animal Model of Sustained Liver Injury and Regeneration Stimulus

**DOI:** 10.1038/s41598-018-32889-y

**Published:** 2018-10-09

**Authors:** Kenta Inomata, Kazuki Tajima, Hiroshi Yagi, Hisanobu Higashi, Hirofumi Shimoda, Kentaro Matsubara, Taizo Hibi, Yuta Abe, Hanako Tsujikawa, Minoru Kitago, Masahiro Shinoda, Hideaki Obara, Osamu Itano, Alejandro Soto-Gutierrez, Yuko Kitagawa

**Affiliations:** 1 Department of Surgery, Keio University, Tokyo, Japan; 2 Department of Pathology, Keio University, Tokyo, Japan; 3 Department of Pathology, University of Pittsburgh School of Medicine, Pittsburgh, PA USA

## Abstract

A feasible large animal model to evaluate regenerative medicine techniques is vital for developing clinical applications. One such appropriate model could be to use retrorsine (RS) together with partial hepatectomy (PH). Here, we have developed the first porcine model using RS and PH. RS or saline control was administered intraperitoneally to Göttingen miniature pigs twice, two weeks apart. Four weeks after the second dose, animals underwent PH. Initially, we tested different doses of RS and resection of different amounts of liver, and selected 50 mg/kg RS with 60% hepatectomy as our model for further testing. Treated animals were sacrificed 3, 10, 17 or 28 days after PH. Blood samples and resected liver were collected. Serum and liver RS content was determined by Liquid Chromatograph-tandem Mass Spectrometer. Blood analyses demonstrated liver dysfunction after PH. Liver regeneration was significantly inhibited 10 and 17 days after PH in RS-treated animals, to the extent of 20%. Histological examination indicated hepatic injury and regenerative responses after PH. Immunohistochemical staining demonstrated accumulation of Cyclin D1 and suppression of Ki-67 and PCNA in RS-treated animals. We report the development of the first large animal model of sustained liver injury with suppression of hepatic regeneration.

## Introduction

Great strides have been made recently in approaches to regenerative medicine. Following the report of techniques for inducing pluripotent stem cells in 2007^1^, their large-scale culture and their differentiation into mature hepatocytes^2–4^, expectations have been raised concerning their potential clinical application for treating end-stage liver disease. In order to prove the feasibility and usefulness of this technology, pre-clinical large animal models are absolutely necessary. However, these are yet to be established, representing the most serious obstacle to translation to the human clinical setting.

A wide variety of liver injury models has been established in rodents^5–7^, and these methodologies have been tested in large animals too. However, a majority of these liver failure models is characterized by acute hepatic injury. Acetaminophen and D-galactosamine drug-induced toxicity have been widely used to mimic fulminant hepatitis and successfully adapted to large animals like pigs. These models demonstrated acute hepatic injury and high mortality^8,9^. Additionally, surgical models using hepatectomy or devascularization have also been reported in both rodents and large animals^10–13^. In these latter models, also with high mortality, the optimal extent of liver resection to test regenerative strategies is still unclear^11,12^. Although a small-for-size transplantation model in pig has been reported^14,15^, this model also showed a high mortality rate and it was still difficult to determine the volume of transplanted liver graft. To evaluate coagulation potential as an aspect of liver function, a hemodilutional coagulopathy model with blunt liver injury was also reported^16,17^. However, this model only allowed observation for a few hours and could thus only evaluate short-term outcomes such as coagulation parameters or blood loss. Hence there remains an urgent need for animal models of chronic liver disorders, particularly to develop regenerative medicine approaches. To evaluate regenerative therapies using iPS/ES cells, a sufficient period of time for assessing engraftment and cell viability after introduction of stem cells is required. In an attempt to accomplish this, a drug-induced chronic liver injury model in pigs was developed. Chronic liver failure with fibrosis mimicking liver cirrhosis was triggered by carbon tetrachloride. This model is indeed useful for regenerative medicine studies, but the simple administration of such a chemical results in acute toxicity and large inter-individual differences^18^. Other models have also been reported, including knocking out specific metabolic systems, such as fumarylacetoacetate hydrolase (FAH) in the pig^19,20^. FAH-deficient pigs exhibit acute and sub-chronic liver injury, but suffer the disadvantage that genetic modifications and breeding programs can only be accomplished in specialized institutions. Therefore, this type of model lacks general accessibility, although undoubtedly valuable for studies of regenerative medicine.

In other approaches, for acute and chronic liver injury models, hepatic regeneration processes require maintenance of regenerative signals induced by the hepatic injury. On the other hand, transplanted cells need a functional advantage over the remaining host cells in order to repopulate efficiently. Although the extensive liver injury caused in all the earlier models does accelerate the regenerative signals for the remnant liver, transplanted cells are commonly less robust than the recipient’s own hepatocytes and tend to decline over time.

A more appropriate model for applications in pre-clinical regenerative medicine is the use of retrorsine (RS) together with partial hepatectomy (PH), as established in rats^21,22^. RS is a pyrrolizidine alkaloid that blocks the cell cycle and triggers hepatocyte senescence^23,24^. In this rat model, the ability of endogenous hepatocytes to proliferate following PH is decreased by RS. However, no large animal model using RS has been reported and the distribution of RS through the body in large animals after injection is not known.

Thus, the aim of the present study was to develop the first large animal model, characterized by sustained liver injury with suppression of hepatic regeneration, and to describe the clinical, biochemical and histological features of this model. The suppressed hepatocellular regeneration in this model should be useful for pre-clinical studies of regenerative therapies.

## Results

### Standardization of procedures for 60% hepatectomy combined with RS administration in pigs

**Figure 1:**
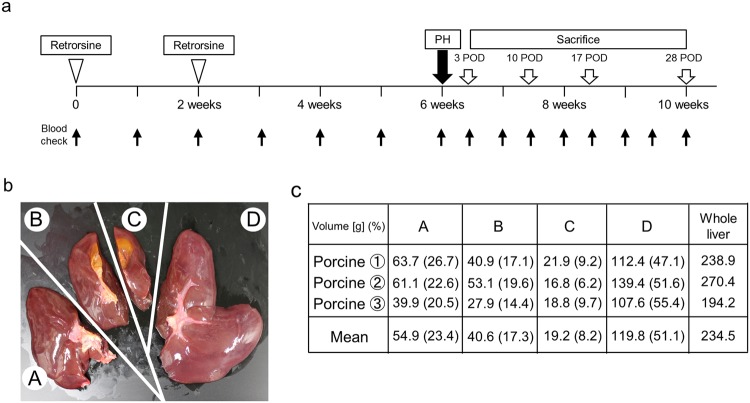
The time course and representative images of protocol segments. (**a**) Time course of retrorsine administration, partial hepatectomy and sacrifice. (**b**) Bench dissection of pig liver. (**c**) Ratio of partial weight of dissected liver.

First, we standardized the protocol for RS treatment and PH (Fig. 1a) [Time line of the experiment: Pigs were treated twice with RS via an intraperitoneal catheter two weeks apart followed by PH and sacrifice on day 3, 10, 17 or 28 after PH]. Preliminary *ex vivo* dissection of three cadaveric porcine livers was performed to normalize the resection technique and to calculate segmental volumes (Fig. 1b). As shown in Fig. 1b, we divided the pig livers into four parts. The right lateral lobe was labeled “A”. We additionally divided the right medial lobe along the line of the gall bladder bed. Then the right side of the right medial lobe was labeled “B” and the left side of the right medial lobe was labeled “C”. The left medial and left lateral lobe were labeled “D”. The resected parts of the liver, composed of A, B, C and D constituted 20.5–26.7% (mean 23.4%), 14.4–19.6 (mean 17.3%), 6.2–9.7% (mean 8.2%) and 47.1–55.4% (mean 51.1%) of the total liver volume, respectively (Fig. 1c). The right side of the liver was chosen as the ideal resection remnant because it contains the inferior vena cava within the parenchyma. Thus, residual liver weight was almost 60% when C and D were resected. Those animals receiving B, C and D resection (almost 80% resection) died of liver failure within 10 days, while those with C and D resection or only D resection survived long enough for their livers to be harvested at the times noted above. Therefore, C and D resection was chosen as the ideal resection procedure. Technically, the anatomical resection could be reliably performed and the intraperitoneal catheter was safely inserted in all pigs.

### Injection of 50 mg/kg of RS is feasible in this porcine model

**Figure 2:**
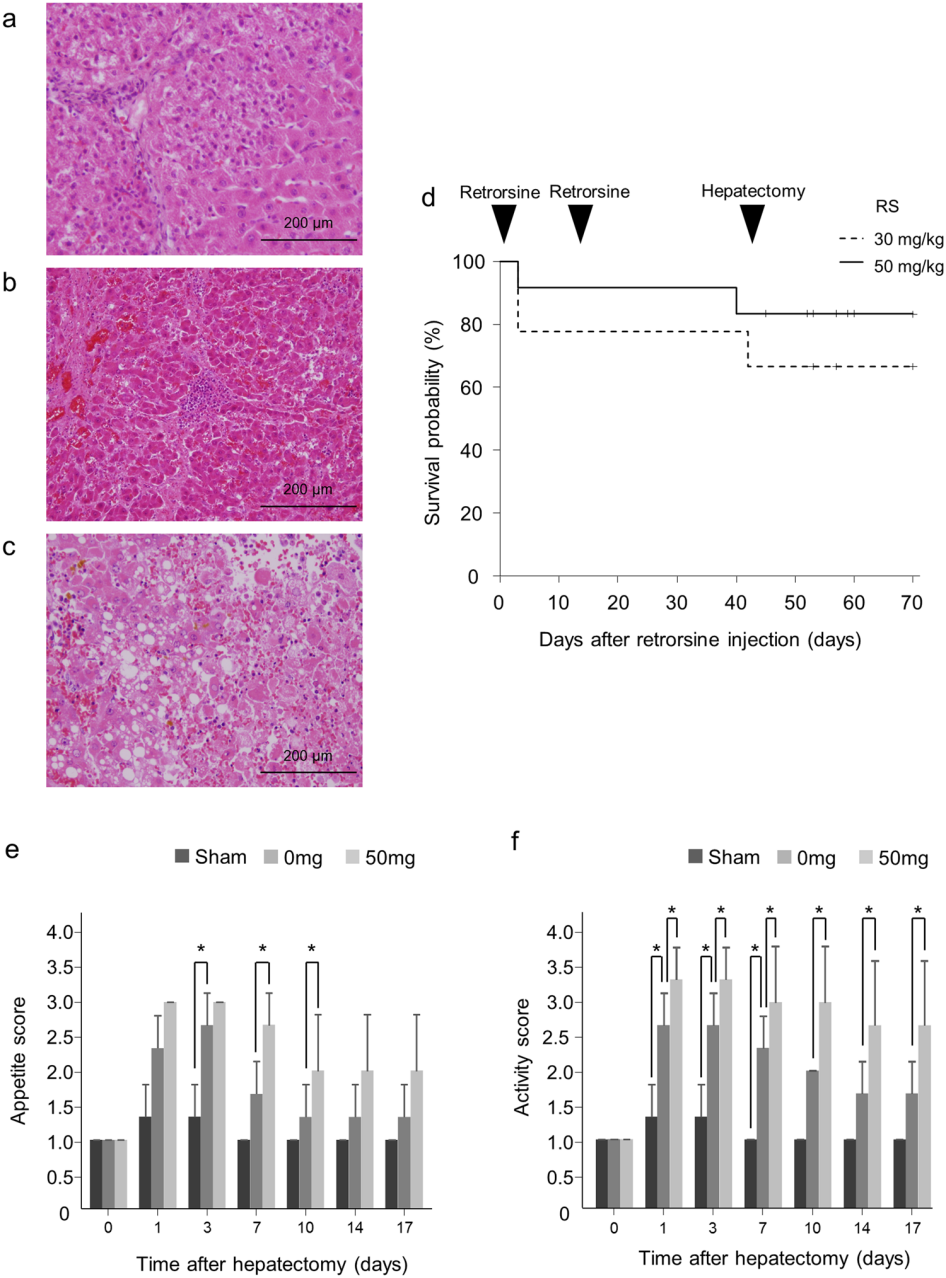
Clinical outcome in animals given retrorsine. (**a**–**c**) H & E staining of liver from (**a**) a pig receiving 30 mg/kg RS, dying 3 days after the first RS injection, (**b**) a pig receiving 50 mg/kg RS, dying 26 days after the second RS injection and (**c**) a pig receiving 100 mg/kg RS, dying on the day after the second injection of RS. (**d**) Kaplan-Meier survival curves for follow-up of 21 pigs after the first injection of RS. (**e**,**f**) Clinical scores of appetite (**e**) and activity (**f**) of pigs after 0 or 50 mg/kg RS treatment (mean + SD). ^*^P < 0.05.

This experimental model, inducing liver failure by combining surgical treatment with pharmacological treatment, was well-reproducible in the present study. Preliminary investigations were undertaken using titrated doses of 10, 30 and 50 mg/kg RS with monitoring of the effects 3, 10, 17 and 28 days after PH. In total, we injected RS into 24 pigs (10, 30, 50 and 100 mg/kg into 2, 9, 12 and 1 animal, respectively). Six animals died after RS injection. Of these six animals, two injected with 30 mg/kg RS and one with 50 mg/kg RS had obviously raised blood levels of AST (>1000 IU/L), alanine transaminase (ALT) (>100 IU/L) and prothrombin time (PT) (>35 s). These three animals died 3 days after the first injection. The liver was atrophic in the autopsy and histological examination demonstrated fatty and vacuolar degeneration of hepatocytes which suggested acute hepatic injury (Fig. 2a). Another animal which was injected with 30 mg/kg RS also showed liver dysfunction according to laboratory data of the blood. Ammonia (NH3) concentration was increased and PT prolonged; it died with hematemesis 42 day after the first injection. One animal which died 40 days after the first injection of 50 mg/kg RS also showed abnormal liver function on laboratory data and vomiting the day after the first injection; PT was prolonged and the animal died with bleeding of the nose. There were bloody ascites in the autopsy and histological examination showed widespread hemorrhagic necrosis in liver tissue (Fig. 2b). It was hypothesized that these two animals was died with gastrointestinal bleeding due to the tendency to bleed resulting from hepatic insufficiency. We also treated one pig with 100 mg/kg RS, which resulted in symptoms like vomiting and loss of appetite from the time of the first injection. Laboratory data of the blood temporary improved but we sacrificed the animal on the day after second injection because PT was prolonged again and body weight, activity or appetite was not recovered. Its liver surface was rough and liver tissue showed obvious atrophic signs (135.6 g). Microscopically, there was severe necrotic change (Fig. 2c). All untreated pigs undergoing 60% PH survived up to the time of sacrifice. As shown in Fig. 2d, the survival rate of pigs treated with 50 mg/kg RS (83.3%) was higher than in those treated with the lower dose of 30 mg/kg RS (66.7%). We evaluated the effect of RS on clinical features in these animals. In animals either treated or not treated with RS, both appetite and activity scores were higher than in sham-operated animals, with the clinical scores tending to be higher in RS-treated animals than controls. (Fig. 2e,f).

**Figure 3:**
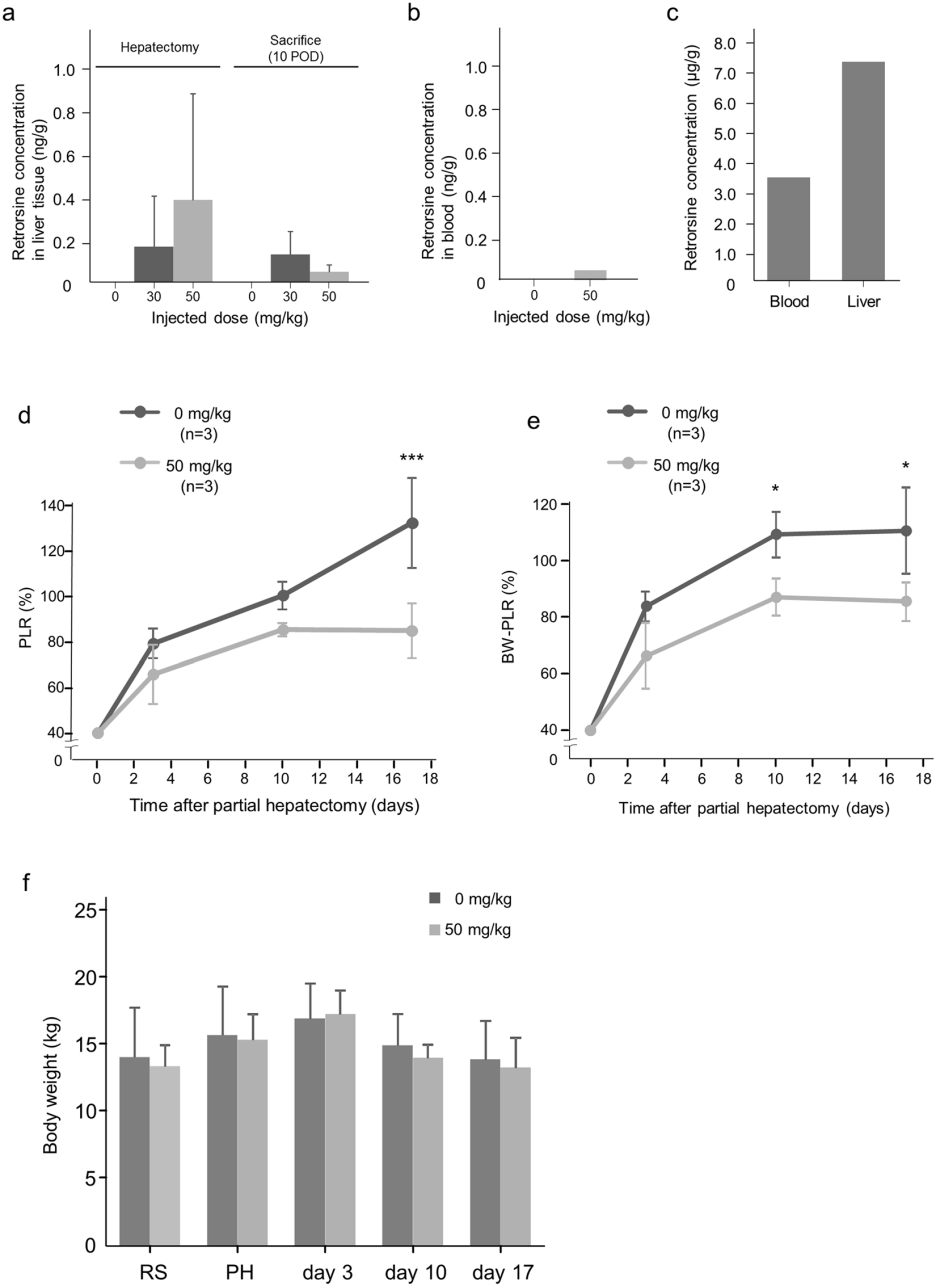
Retrorsine concentration in liver tissue and blood and liver regeneration in animals after hepatectomy with/without retrorsine. (**a**) RS concentration in liver tissue of animals which received 30 and 50 mg/kg RS and control animals at the time of PH and 10 day post-PH (mean + SD). There was no significant difference among the groups. (**b**) RS concentration in blood of animals which received 50 mg/kg RS at the time of 7 days after second injection of RS. The significance of difference was not able to be evaluated due to the small number of samples. (**c**) RS concentration in blood and liver tissues from a pig exposed to 100 mg/kg RS. (**d**) PLR of in pigs exposed to 0 mg/kg or 50 mg/kg RS whose liver contained 0.0477 ng/g RS on day 10 after PH (mean + SD). ^***^P < 0.001. (**e**) BW-PLR in pigs exposed to 0 mg/kg or 50 mg/kg RS (mean + SD). ^*^P < 0.05. (**f**) Body weight of pigs exposed to 0 mg or 50 mg RS (mean + SD).

RS was detected by Liquid Chromatograph-tandem Mass Spectrometer in liver tissues from RS-treated pigs at the time of PH (28 days after the last injection) and even 10 days after PH (Fig. 3a). In contrast, minimal RS was detected in the blood a week after the final injection, and was close to the limit of detection (Fig. 3b). The RS concentration was extremely high both in blood and liver tissues from a pig injected with 100 mg/kg RS 15 days after the first injection (Fig. 3c).

### RS suppresses regeneration of the remnant liver

After PH, the remnant liver is usually enlarged as a response to injury. In the present study, the volume of the remnant liver in RS-treated animals was smaller than in the controls throughout the 17 day period after PH. Three days after PH, liver regeneration in RS-treated animals amounted to only 60% of estimated whole liver volume, while it was already 80% in controls (Fig. 3d). These differences between the RS-treated and control groups in Percentage of liver regeneration (PLR) 17 days after hepatectomy were statistically significant (*P* value = 0.0008) (Fig. 3d). The body weight-adjusted percentage of liver regeneration (BW-PLR) showed a similar tendency and was significantly lower in the RS-treated group 10 and 17 days after hepatectomy (*P* value were 0.0215 and 0.0426, respectively) (Fig. 3e). For comparison, we performed PH in pigs after the administration of 30 mg/kg of RS, and found that liver volumes of 30 mg/kg RS-treated animals recovered to the same degree as in control animals at day 28. Conversely, results on the PLR of a single 50 mg/kg RS-treated animal was 81.8% and indicated that recovery might be suppressed to 80% on day 28 after PH. Therefore, it is possible that inhibition of regenerative potential by 50 mg/kg persisted at least 28 days after PH, although more cases are needed to confirm this. Body weight of animals had not changed significantly from the first injection of RS to 17 days after PH and there was no significant difference in body weight of control and RS-treated animals (Fig. 3f).

### RS causes sustained hepatic injury

**Figure 4:**
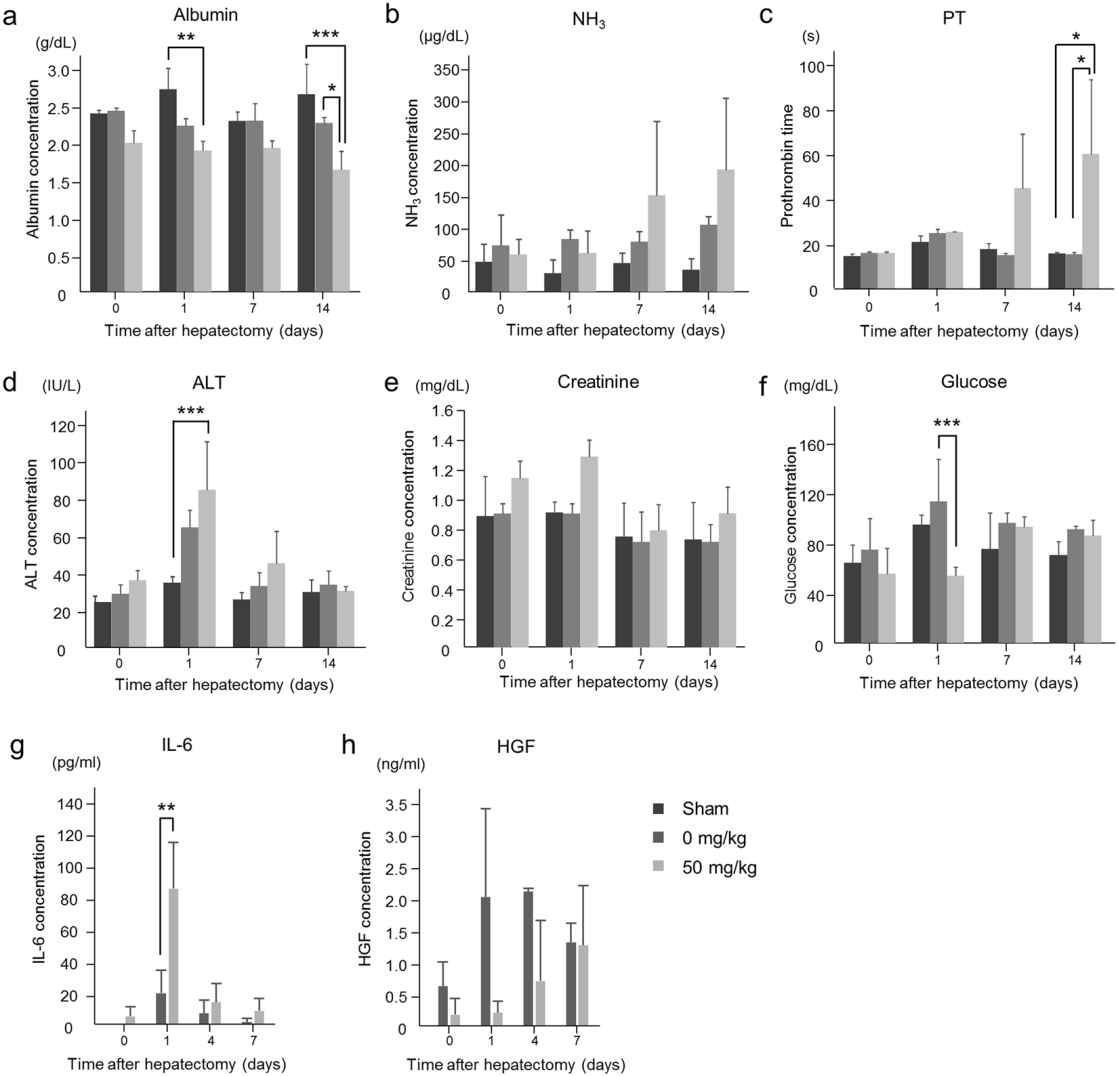
Blood biochemistry (**a**–**h**) Concentrations of albumin (**a**), NH3 (**b**), PT (**c**), ALT (**d**), creatinine (**e**), glucose (**f**), IL-6 (**g**) and HGF (h) in blood of control and RS-treated pigs (Mean + SD). ^*^P < 0.05, ^**^P < 0.01 and ^***^P < 0.001.

Blood tests demonstrated abnormalities in several parameters of liver function after PH in RS-treated animals (Fig. 4) in that albumin levels continuously declined and were significantly lower than in sham-operated animals the day after PH and untreated animals 1 and 14 days after PH (Fig. 4a). Plasma NH3 levels tended to be higher than in controls and sham-operated animals (Fig. 4b). The PT of RS-treated animals was durably prolonged and was longer than in sham-operated and control animals 14 days after PH (Fig. 4c). The ALT concentration was increased on the first day after PH and gradually decreased to normal levels thereafter (Fig. 4d). Both the ALT concentration and serum creatinine levels of RS-treated animals tended to be higher than in controls (Fig. 4d,e), whereas blood glucose was lower (Fig. 4f). Plasma Interleukin-6 (IL-6) concentration was significantly higher in RS-treated animals than controls on the first day after PH, and hepatocyte growth factor (HGF) concentration tended to be higher 1 to 4 days after PH, as documented by Enzyme-Linked Immuno-Sorbent Assays (ELISA) (Fig. 4g,h).

### PH triggers regenerative responses in the remnant liver but RS affects this by suppressing cell division

**Figure 5:**
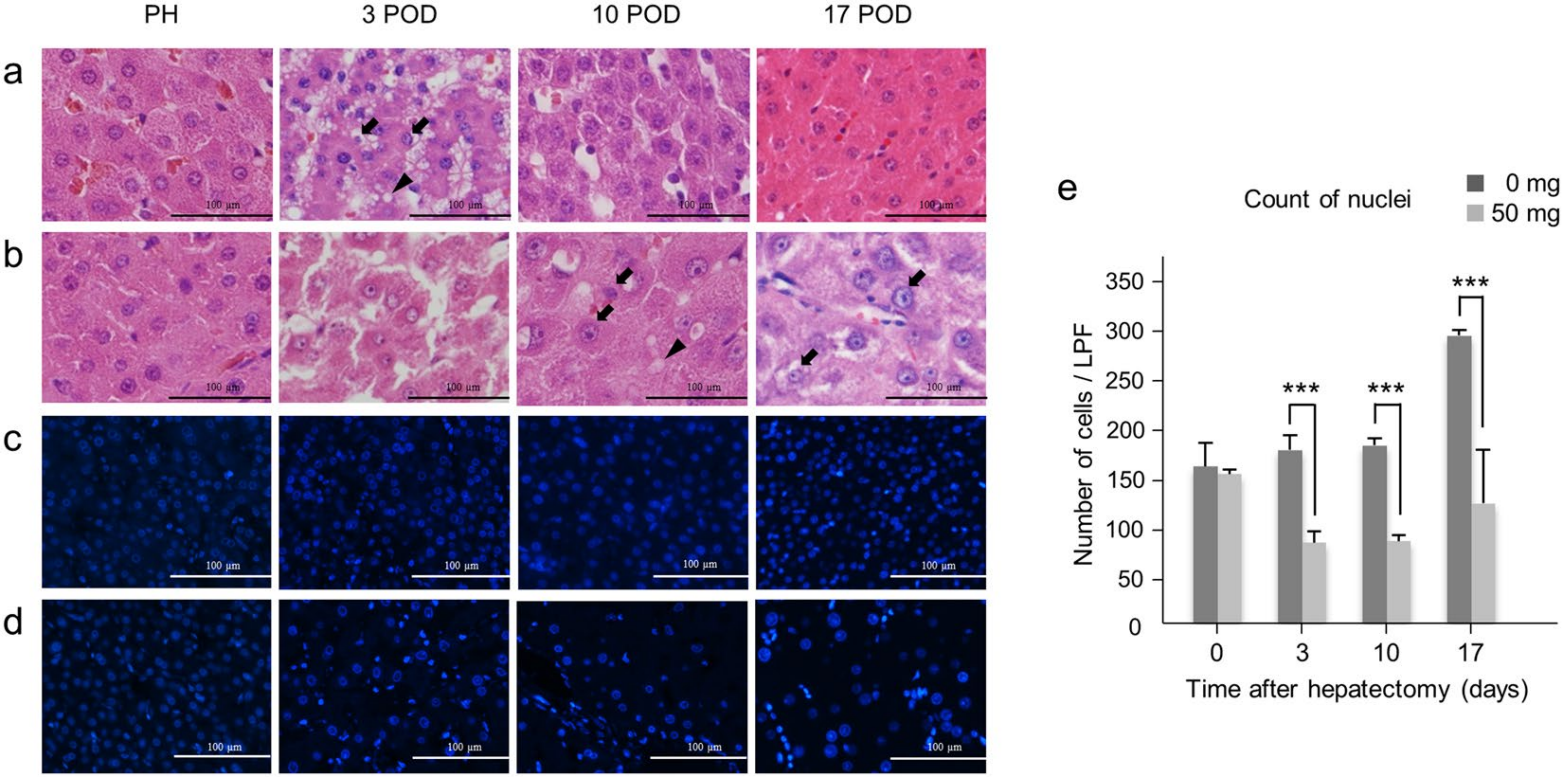
Liver histology after hepatectomy. (**a**) H & E staining of control liver tissue. Anisokaryosis (arrow) and fatty degeneration (arrow head). (**b**) H & E staining of liver tissue from a 50 mg/kg RS-treated pig. (**c**,**d**) Immunohistochemical staining of 4′,6-diamidino-2-phenylindole in liver tissue from a control pig (**c**) and 50 mg/kg RS-treated pig (**d**). (**e**) Number of 4′,6-diamidino-2-phenylindole-positive cells (mean + SD). ^***^P < 0.001.

**Figure 6:**
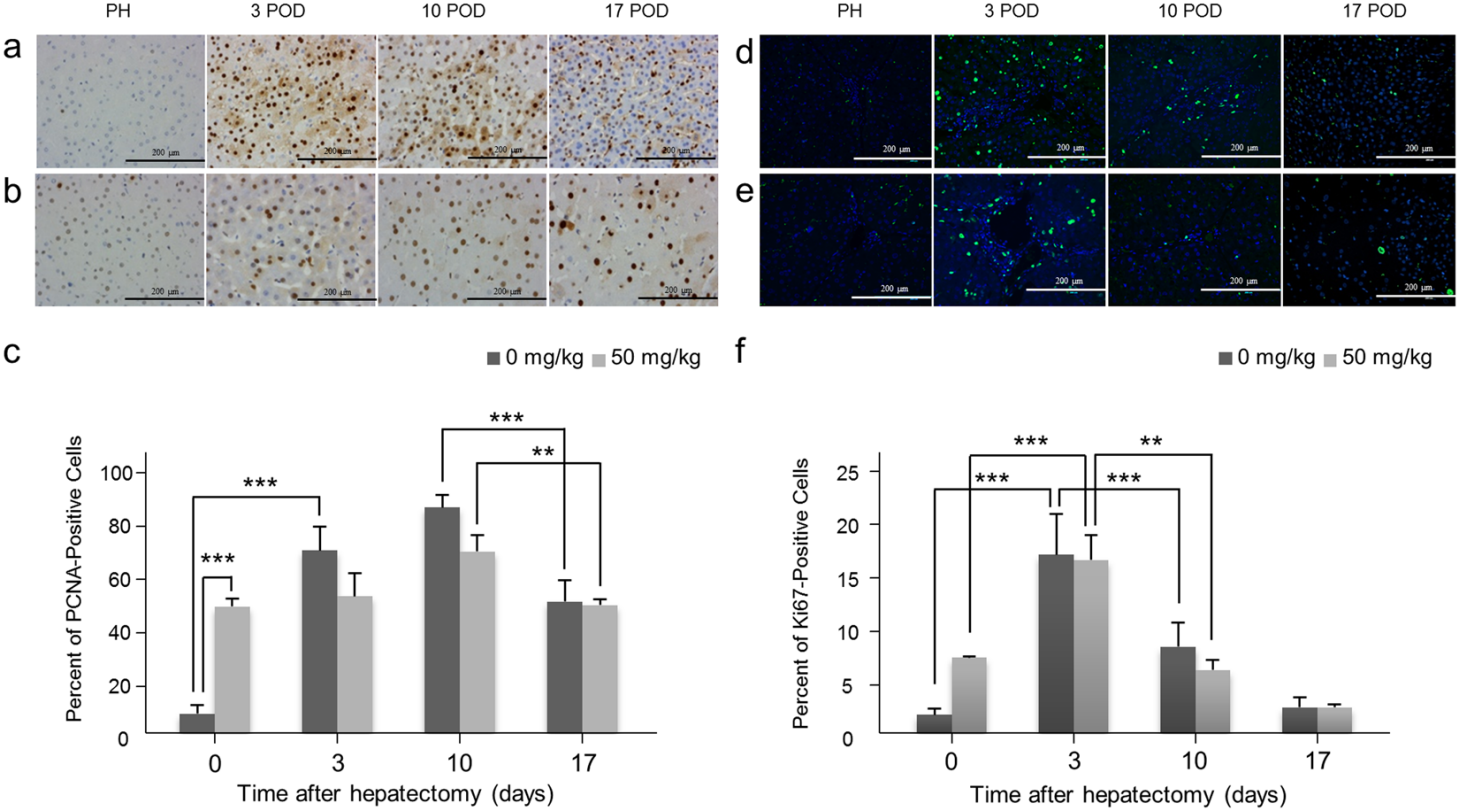
PCNA and Ki-67 staining of liver treated with retrorsine and control. (**a**,**b**) PCNA staining of liver tissue from a control pig (**a**) and a 50 mg/kg RS-treated pig (**b**). (**c**) Percentage of PCNA-positive cells (mean + SD). **P < 0.01 and ^***^P < 0.001. (**d**,**e**) Immunohistochemical staining of Ki-67 in liver tissue of a control pig (**d**) and a 50 mg/kg RS-treated pig (**e**). (**f**) Percentage of Ki-67-positive cells (mean + SD). ^**^P < 0.01 and ^***^P < 0.001.

Hematoxylin-eosin (H & E) staining demonstrated mixed small and large hepatocyte nuclei, and accumulations of fatty globules in hepatocytes following PH; this anisokaryosis and simultaneous fatty degeneration indicates both regenerative and degenerative changes in the hepatocytes^25,26^. These effects were detected 10 and 17 days after PH in RS-treated animals, while they were already present after only 3 days in controls (Fig. 5a,b). By 4′,6-diamidino-2-phenylindole (DAPI) staining, the number of nuclei was significantly decreased in RS-treated animals following PH, whereas in controls immediately after PH, nuclei had increased by 3 days and remained higher through day 17 (Fig. 5c–e). To gain further insights into the regenerative response in pigs exposed to RS, we next quantified the proliferating cell nuclear antigen (PCNA) and Ki-67 expression^25,27,28^. The number of PCNA-positive cells was significantly higher in RS-treated animals than controls at PH. However, it was lower than in controls after PH, although this difference was not significant. The number of PCNA-positive cells peaked in both groups 10 days after PH (Fig. 6a–c). Ki-67 was also over-expressed 3 days after PH in both groups but nonetheless tended to be lower in RS-treated animals, although also not reaching statistical significance (Fig. 6d–f).

### Effects of RS treatment include the accumulation of Cyclin D1 in liver tissue and a delayed peak of apoptosis

**Figure 7:**
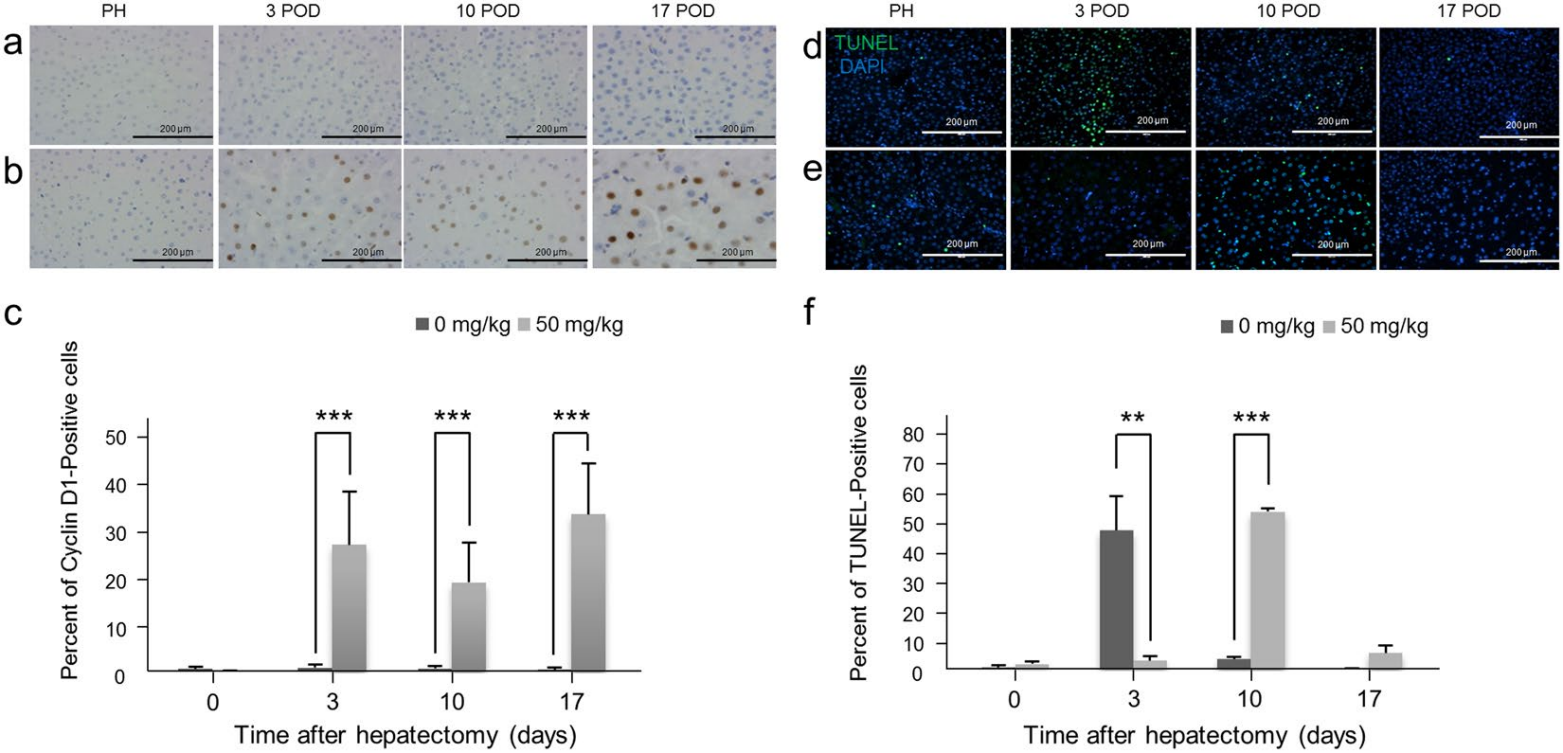
Cyclin D1 and TUNEL staining of the livers after hepatectomy. (**a**,**b**) Cyclin D1 staining of liver tissue of a control pig (**a**) and a 50 mg/kg RS-treated pig (**b**). (**c**) Percentage of cyclin D1-positive cells (mean + SD). ^***^P < 0.001. (**d**,**e**) TUNEL staining of liver tissue of a control pig (d) and 50 mg/kg RS-treated pig (**e**). (**f**) Percentage of TUNEL-positive cells (mean + SD). ^**^P < 0.01 and ^***^P < 0.001.

Cyclin D1, which is associated with G1-S phase transition in the cell cycle, is generally over-expressed in cells that cannot enter S phase^29,30^. Consistent with this, significant over-expression of cyclin D1 was detected in hepatocytes following PH in the RS-treated animals. On the other hand, the expression of cyclin D1 in control animals remained at low levels even after PH (Fig. 7a–c). The TdT-mediated dUTP nick end labeling (TUNEL) staining performed to detect apoptotic hepatocytes showed that the percentage of TUNEL-positive cells was already increased 3 days after PH in control animals, and was decreased again after 10 days, whereas in treated animals it was significantly increased 10 days after PH (Fig. 7d–f). Western blotting demonstrated over-expression of cyclin D1 in the liver of RS-treated animals after PH, while Caspase-3 was not detected in the liver of control or treated animals (Supplementary Fig. S1–4).

### Inter-individual differences in sensitivity to RS

There were clear inter-individual variations in RS sensitivity. Two of 9 pigs receiving 30 mg/kg RS were unable to eat or walk after the first injection, and plasma AST and ALT elevation and prolonged PT was worse than in other animals which were uneventful until sacrifice. Three days after the first injection of RS, their activity decreased further and they died. The liver was atrophic at autopsy and fatty and vacuolar degeneration of hepatocytes was observed by histological examination. One of the 50 mg/kg RS-treated pigs lost appetite and vomited a day after PH. Although plasma AST and ALT were elevated and PT was prolonged temporarily, laboratory data gradually improved. However, slight AST and ALT elevations were sustained and PT become prolonged again and eventually the animal died with bleeding from the nose 26 days after the second injection. Bloody ascites was found at autopsy and histological examination showed widespread hemorrhagic necrosis in liver tissue. Although no ulcers were seen in the stomach, we suggest that the cause of death was gastrointestinal bleeding associated with severe hepatopathy. One pig treated with 100 mg/kg RS suffered vomiting and loss of appetite from the time of the first injection. Blood tests on the day following the first injection of 100 mg/kg RS showed extremely high levels of AST, ALT and PT and indicated severe liver dysfunction (Supplementary Table. S1). Though AST and ALT concentration and PT decreased over time, clinical findings such as body weight, activity and appetite did not recover and PT began to increase again. We decided to sacrifice the animal 15 days after the first injection of RS. Its liver surface was rough and the organ was obviously atrophic at autopsy; microscopically, there was severe necrotic change.

## Discussion

To evaluate the feasibility and functionality of transplanted cells, such as isolated primary cells or ES/iPS cell-derived hepatocytes, the major limitations of the thus-far reported large animal models have been 1) acute liver failure resulting in high mortality, and 2) no selective growth advantage for transplanted cells over host hepatocytes for effective repopulation. A certain period of time is required before any transplanted cells can be evaluated for their expansion and regeneration capacity *in vivo*; thus, several acute liver failure models such as those using hepatectomy or drug toxicity have a high mortality rate in the acute phase^8,11,31^ and may not provide a sufficient time window for assessing regenerative capacity. Additionally, using hepatectomy or hepatotoxins as the regenerative stimuli to observe repopulation by transplanted cells in these models may not imbue the latter with a sufficient growth advantage over the host hepatocytes outnumbering them. In contrast, the RS + PH model reported in rats appeared to overcome these limitations in that the suppression of hepatocyte regeneration was shown by loss of liver volume and repopulation of transplanted cells using the DPP4 transgene^22^. Therefore, RS has been widely used for regenerative research in rodents, but has not been successfully adapted to large animal models. This may be partly because of differences in sensitivity and *in vivo* kinetics or differences in the distribution of RS in the body. Hence, to the best of our knowledge, the present study is the first to report details of a large animal model using RS in pigs, and tracking its concentration in the blood and liver after intraperitoneal injection. We showed that RS was retained in liver tissue but not in the blood of these animals after PH was performed. This distribution is consistent with the notion that RS has a role in suppressing liver regeneration after PH in pigs, as previously reported. Although factors predicting the effect, or the mechanism of hypersensitivity, have not been identified, we have determined some aspects of RS pharmacokinetics in pigs.

As previously reported, combined drug-induced and surgical models for acute liver failure cause high mortality with short survival periods of only 3–48 h^8,9,11,32^. Moreover, it proved to be difficult to determine the ideal extent of hepatectomy, because small resections had no impact due to the large capacity of the liver to regenerate, while extensive resections of up to 80% of the whole organ increased the risk of fatal liver failure^11,12^. In the present study, we determined 60% resection to be a safe extent of resection in anticipation of the RS effect, and found that this surgical model was reproducible. On the other hand, it is also well known that determining feasible doses of hepatotoxic drugs is difficult^33,34^. Interestingly, 30 mg/kg of RS was more toxic than 50 mg/kg and resulted in worse survival. Considering that the body weight of individuals who died before PH tended to be lower than other animals at the moment of first RS injection, the condition of the animals before RS treatment might have had an effect on toxicant sensitivity and survival. Additionally, it cannot be excluded that there might have been differences in individual animal sensitivity to the drug. Although there were some individuals showing extreme sensitivity to RS in this study, the mortality rate of 50 mg/kg RS-treated animals followed by 60% liver resection was lower than in other large animal models^8,18,35^ and liver regeneration was successfully suppressed in pigs injected with 50 mg/kg RS. Therefore, we suggest that this dose of RS together with 60% liver resection is feasible for producing a reproducible model.

This type of drug induction and PH model yielded clinical symptoms and abnormalities in the laboratory data as appropriate for studying regenerative capacity. The clinical score for both appetite and activity indicated that the RS-treated animals were in a worse condition than controls during the observation period, and there were some abnormalities in blood values. Low level serum albumin and prolonged PT suggested that diminished protein synthesis and a tendency toward increased NH3 concentrations might be caused by the decreased detoxification capacity of the liver. Due to small sample size, significant differences were shown only for some of the data. Nonetheless, there were tendencies towards several abnormalities in biochemical parameters throughout the follow-up period. Thus, a worsening of the general condition of the animal caused by liver dysfunction, reduced protein synthesis and liver detoxification may provide the opportunity to evaluate interventions to correct these deficits.

In the present study, we describe the time course of liver regeneration in pigs. The recovery of liver volume was suppressed in RS-treated animals throughout a 17 day period after PH. The first 3 days after PH were an especially active phase of liver regeneration as shown in PLR and BW-PLR graphs; RS suppressed this recovery of liver volume in that phase. Moreover, one RS-treated animal demonstrated that PLR was suppressed to 81.8% 28 days after PH. Therefore, it is possible that inhibition of regenerative capacity persisted up to 28 days after PH, although more cases are needed to confirm this.

Histological findings also demonstrated that the regenerative responses of RS-treated animals were suppressed 3 days after PH. Although it is difficult to distinguish regenerative reactions from degenerative changes of liver tissues morphologically (because these changes are frequently seen simultaneously), the presence of anisokaryosis and binucleated hepatocytes suggested regenerative responses as well as the expected fatty degeneration^25,26^. Immunohistochemistry data supported the hypothesis that RS-treatment suppressed the cell cycle and delayed liver regeneration. DAPI staining demonstrated that the number of nuclei per unit liver volume was lower in RS-treated animals than controls, and that these nuclei were enlarged and swollen, with cell cycling and hepatocyte division blocked. On the other hand, PCNA expression, marking dividing cells, was not significantly suppressed in RS-treated animals, although the number of PCNA-positive cells was lower in RS-treated animals than controls after PH. In this respect, our pig model seems different from the rat model in that over-expression of PCNA in RS-treated rats has been reported. There were no significant differences between RS-treated animals and controls in the number of Ki-67-positive cells either. In the present study, neither PCNA nor Ki-67 expression was significantly suppressed in RS-treated animals after PH, but very few studies on the expression of these molecules in the liver of large animals have been reported and their role in liver regeneration is unknown. The overexpression of PCNA and Ki-67 peaked around 10 days after PH, which is the time of hepatocyte regeneration of liver volume; we therefore hypothesize that their expression is associated with liver regeneration.

It is well known that both hepatocyte regeneration and apoptosis are upregulated following liver injury and that this requires a variety of cytokines^36^. Cyclin D1 is one of the regulatory proteins of the cell cycle^29,30^. Several studies demonstrated that cyclin D1 accumulates when cycling is suppressed by pyrrolizidine alkaloids such as RS, because of replicative arrest^23,29^. In the present study, overexpression of cyclin D1 was observed in the liver of RS-treated animals after PH. The number of cyclin D1-positive cells was significantly greater in RS-treated animals relative to controls after PH, as previously reported in rats^29^. Furthermore, Western blotting also demonstrated over-expression of cyclin D1 in RS-treated pigs after PH. In terms of the data from the TUNEL assay, the number of apoptotic cells peaked on day 10 after PH in RS-treated animals, while they peaked on day 3 in controls. This suggests that the peak regeneration and apoptosis reaction phase was delayed in RS-treated animals. Western blotting of caspase-3 was performed to evaluate apoptosis of hepatocytes but the expression of this enzyme was not detected in control or RS-treated animals. We speculate that the total content of caspase-3 is very low and even in the control animals, therefore difference between RS-treated animals and controls could not be unequivocally evaluated.

Biochemical and histological data demonstrated that RS remained present in the liver tissue and continued to suppress regeneration after PH. Moreover, the presence of sustained liver injury was reflected in several parameters of blood values and clinical state. This large animal model should be useful for studying transplanted cells on the human scale but in the same manner as in the rat model, and should be valuable for evaluating how transplanted cells or liver grafts correct functional liver disorders. The RS-treated rat model was in fact used to evaluate auxiliary liver transplantation^37^.

In conclusion, we report the creation of the first large animal model of RS and 60% PH characterized by sustained liver injury with suppression of hepatic regeneration. We believe that it is a promising model for improving preclinical studies of liver regenerative medicine.

## Materials and Methods

### Animals

Thirty four female Göttingen miniature pigs weighing 14–20 kg were used. The pigs were bred and maintained in a colony by Oriental Yeast Co. Ltd., Tokyo. The original breeders were obtained from the Ellegaard company in Denmark. The experiments and procedures described here were carried out in accordance with the Guide for the Care and Use of Laboratory Animals as adopted and promulgated by the National Institutes of Health, and were approved by the Animal Care Committee of KEIO University.

### RS administration and PH

Animals were kept under standard laboratory conditions. They were fed a standard diet and fasted for 12 h before surgery. All the experiments were conducted according to local institutional guidelines for the care and use of laboratory animals. 7-month-old Göttingen miniature pigs were assigned to RS treatment (n = 24) or vehicle-treated controls (n = 11) at the outset of the experiment. Pigs in these groups received intraperitoneal RS at 10, 30, 50 or 100 mg/kg (2, 9, 12 and 1 animals, respectively) or vehicle alone (an equal volume of 150 mmol/L saline solution) two weeks apart. The RS solution was prepared as follows: RS (12,18-dihydroxysenecionan-11,16-dione; RS-Longilobine, Sigma, St. Louis, MO) was added to distilled water at 5 mg/ml and titrated to pH 2.0 with 1 N HCl to completely dissolve the solid. Subsequently, the solution was neutralized using 1 N NaOH and was then used immediately. Four weeks after the second RS or vehicle treatment, PH was performed as follows: Pigs in the RS/PH and control/PH groups were euthanized and livers harvested at 3, 10, 17 or 28 days after PH. At the time of PH and each endpoint, liver tissues were obtained and liver weights were recorded. Liver tissue was fixed in 4% paraformaldehyde and processed routinely for paraffin-embedded sections. In addition, tissue was frozen for estimation of RS concentrations. The time line of the study is shown in Fig. 1a.

### Operative procedure

Premedication was performed with intramuscular dexmedetomidine (0.04 mg/kg) and midazolam (0.2 mg/kg). The animals were then intubated and ventilated with a mixture of nitrous oxide (78%), oxygen (20%), and isoflurane (2%). The anesthetized pigs were placed in the supine position on the operating table. In the first operation, two polyethylene cannulas were inserted into the peritoneal cavity for injection of RS and into the right external jugular vein for blood sampling. In the second operation, 60% PH was performed on all animals after intubation and anesthetizing in the same manner as described above. After opening the abdomen, the liver was freed by dividing the triangular ligaments, the falciform ligament, and all peritoneal attachments, including the small omentum. The left portal vein and left hepatic artery were divided and ligated, followed by parenchymal transection. The abdominal wall was closed in two layers. After discontinuation of anesthesia, the animals were extubated, kept in isolated cages, and warmed with infrared lamps. Saline was administered intravenously throughout the surgical procedure. Ceftriaxon was injected intravenously at the beginning of surgery.

### Clinical assessment

Clinical outcomes were defined by scoring based on the animals’ activity and appetite, as shown in Table 1. Specifically, each pig was scored daily for activity (1 = active and comes closer to people or is interested in something, 2 = almost always sitting but standing up when especially interested in something, 3 = always sitting or lying down unless strongly stimulated by visual or acoustic stimuli and 4 = rarely standing up or always lying down) and appetite (0 = good, 1 = weak, and 2 = none). Body weight (BW) of each pig was also recorded.

**Table 1:** Scoring system for appetite and activity of the pigs.

Appetite	1 = normal
	2 = weak/abnormal
	3 = absent
Activity	1 = well
	2 = almost always sitting
	3 = getting up after visual or acoustic stimuli
	4 = cannot get up

### Blood biochemistry

Venous blood was sampled 1, 7, 14, 15, 21, and 28 days after the first injection of RS and 1, 3, 7, 10, 14, 17, 21, 24, and 28 days after PH. Tests included plasma albumin, ALT, NH3, creatinine, glucose and PT. IL-6 and HGF were quantified by ELISA.

### Measurement of RS by Liquid Chromatography/Fourier Transform Ion Cyclotron Resonance Mass Spectrometry (LC-FTICR-MS)

We measured RS concentrations in liver tissue or blood by LC-FTICR-MS. RS was measured in liver tissue of three animals receiving 30 mg/kg RS, three animals receiving 50 mg/kg RS and in one control animal at the time of PH and 10 days post-PH. We also measured RS concentrations in the blood of animals receiving 50 mg/kg RS, and the control 7 days after the second injection of RS. The concentration of RS in liver and blood of the 100 mg/kg RS-treated animal was measured 15 days after the first injection of RS because it died that day. An Agilent 1200 system (Agilent, http://www.agilent.com) coupled to a LTQ Orbitrap XL (Thermo Fisher Scientific, http://www.thermofisher.com) was used for Liquid Chromatograph-tandem Mass Spectrometer (LC-MS). The data were acquired and browsed using Xcalibur software version 2.1.0 (Thermo Fisher Scientific). A methanol extract was applied to a TSKgel column, ODS-100V (3 mm × 50 mm, 5 µm; TOSOH Corporation, http://www.tosoh.com). Water (LC-MS grade; solvent A) and acetonitrile (LC-MS grade; solvent B) were used as the mobile phase with 0.1% v/v formic acid added to both solvents. The gradient program was as follows: 3% B to 97% B (15 min), 97% B (5 min) and 3% B (5 min). The flow rate was set to 0.4 ml/min, and the column oven temperature at 40 °C; 5 µL of each sample were injected. To monitor HPLC elution, a photodiode array detector was used in the wavelength range 190–950 nm. The Heated ESI setting was as follows: heater temp 400 °C and spray voltage 3.5 kV, and capillary temperature 300 °C for positive-ionization modes. Nitrogen sheath gas and auxiliary gas were set at 50 and 10 arbitrary units, respectively. A full MS scan was performed in the m/z range 100–1500 at a resolution of 60 000 (at m/z 400). Data-Dependent MS/MS fragmentation on the top 4 ions was carried out at a normalized collision energy of 35.0% and an isolation width of 2.0 (m/z), and obtained in ion trap mode.

### PLR and BW-PLR calculation

We measured the weight of “resected liver” at the time of PH and “remnant liver” at sacrifice on a digital scale. On the grounds that resected liver was principally 60% of the whole liver, the volume of the latter at the time of PH was estimated by dividing the resected liver volume by 60/100. To express the degree of recovery relative to the original liver volume at the time of PH, the percentage of liver regeneration is expressed as the ratio of liver volume at the time of sacrifice to estimated whole liver volume at the time of PH. We have termed the liver volume at the time of sacrifice the “remnant liver volume at sacrifice” For this, PLR was calculated using the following formula:

1 $${\rm{PLR}}=\frac{{\rm{remnant}}\,{\rm{liver}}\,{\rm{volume}}\,{\rm{at}}\,{\rm{sacrifice}}\,(g)}{{\rm{resected}}\,{\rm{liver}}\,{\rm{volume}}\,{\rm{at}}\,{\rm{PH}}\,(g)\times (\frac{100}{60})}\times \mathrm{100} \% $$

To consider the body weight of the pig, we added the perioperative BW to the formula. Thus, we defined body weight-adjusted percentage of liver regeneration by the following formula:

2 $$\mathrm{BW}-\mathrm{PLR}=\frac{{\rm{remnant}}\,{\rm{liver}}\,{\rm{volume}}\,{\rm{at}}\,{\rm{sacrifice}}\,(g)/\mathrm{BW}\,{\rm{at}}\,{\rm{sacrifice}}\,(\mathrm{kg})}{{\rm{resected}}\,{\rm{liver}}\,{\rm{volume}}\,{\rm{at}}\,{\rm{PH}}\,(g)/\mathrm{BW}\,{\rm{at}}\,{\rm{PH}}\,(\mathrm{kg})\times (\frac{100}{60})}\times \mathrm{100} \% $$

### Histology

Postmortem examinations were performed in all cases just after death. The macroscopic aspect of the liver was monitored. Specimens were fixed in 4% paraformaldehyde and embedded in paraffin, and sections of 4 µm were cut for staining with H & E. Additional sections were stained to assess Cyclin D1, Ki-67, PCNA and apoptosis using TUNEL method. We also performed DAPI staining to enumerate hepatocyte nuclei. Immunohistochemistry for Ki-67 (Abcam, Cambridge, MA, 100:1), PCNA (Nichirei, Tokyo, Japan, Ready to use) and cyclin D1 (Nichirei, Tokyo, Japan, Ready to use) was performed using specific monoclonal antibodies as primary antibodies. In brief, sections were treated for heat-mediated antigen retrieval in citrate buffer at pH 6.0, and each primary antibody was applied after incubating the sections in blocking reagent. After rinsing, Ki-67-stained samples were incubated with secondary antibody labeled with Alexa 488, and PCNA and Cyclin D1 samples were incubated with peroxidase-labeled antibody. Peroxidase activity was detected with diaminobenzidine (DAB). For TUNEL assays, cells were labeled and stained using the DeadEnd TUNEL System (Promega, Madison, WI, USA).

### Western blotting

Tissue lysis and Western blotting was carried out using the methods described previously^38^. Colon cancer cell line HCT116 was used as the positive control^39^. Rabbit polyclonal antibody against human and pig Caspase-3 (1/200, Anti-Caspase-3 antibody, ab4051 from Abcam, Cambridge, UK), Cyclin D1 (1/2, anti-Cyclin D1 antibody, 413521 from Nichirei, Tokyo, Japan) and GAPDH (1/1000, anti-GAPDH antibody, 10494-1-AP from Proteintech, Illinois, USA) were used for Western blotting.

### Statistical analysis

Statistical analysis was performed using GraphPad Prism version 7.0C for Mac OS X (Graph Pad Software, La Jolla California USA). The mean values for PLR, BW-PLR, serum levels of albumin, ALT, NH3, creatinine, glucose, IL-6, HGF and PT and positive marker staining were compared by two-way ANOVA. The mean values for multiple samples were compared by the multiple comparisons Sidak method. *P* values of <0.05 were considered significant.

## Electronic supplementary material

Supplementary Information

## Data Availability

The datasets generated during or analysed during the current study are available. All data generated or analysed during this study are included in this published article (and its Supplementary Information files).
