# Supplementary material for: A Pre-Clinical Large Animal Model of Sustained Liver Injury and Regeneration Stimulus
Source: Sci Rep. 2018 Oct 9;8:14987. doi: 10.1038/s41598-018-32889-y (PMC6177392; doi:10.1038/s41598-018-32889-y)
Supplement: Supplementary file 1 — Supplementary Information [file 41598_2018_32889_MOESM1_ESM.pdf]

A Pre-Clinical Large Animal Model of Sustained Liver Injury and Regeneration Stimulus

Kenta Inomata†, Kazuki Tajima†, Hiroshi Yagi*, Hisanobu Higashi, Hirofumi Shimoda, Kentaro Matsubara, Taizo Hibi, Yuta Abe, Hanako Tsujikawa, Minoru Kitago, Masahiro Shinoda, Hideaki Obara, Osamu Itano, Alejandro Soto-Gutierrez, Yuko Kitagawa

†Dr. Inomata and Dr. Tajima contributed equally to this work.

Contents:

Supplementary Table S1

Supplementary Figure S1

Supplementary Figure S2

Supplementary Figure S3

Supplementary Figure S4

	100 mg/kg RS-treated pig	Control
ALB (g/dL)	2.5	4.4 ± 1.4
NH3 (μg/dL)	93	43.7 ± 20.3
PT (s)	54.2	19.6 ± 1.5
T.bil (mg/dL)	1.0	0.39 ± 0.43
ALT (IU/L)	455	37.0 ± 2.4
AST (IU/L)	10597	71.3 ± 5.6
ALP (IU/L)	170	583.7 ± 241.2
GTP (IU/L)	66	213.7 ± 178.2

Supplementary Table. S1. Plasma factors in a 100 mg/kg RS-treated pig. Liver functional markers in a pig on the next day of 100 mg/kg RS administration and in control animals to which saline solution was administered (mean ± SD). Abbreviations; ALB, albumin; NH3, ammonia; PT, prothrombin time; T.bil, total bilirubin; ALT, alanine amino transferase; AST, aspartate amino transferase; ALP, alkaline phosphatase; GTP, Glutamyltranspeptidase.

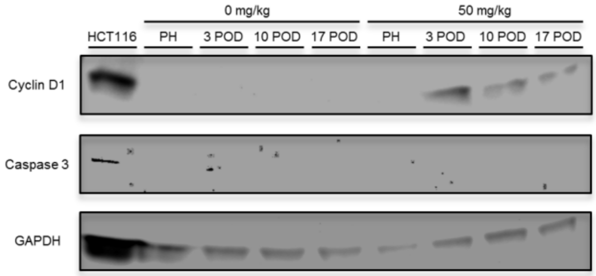

Supplementary Figure. S1. Western Blot analysis of Cyclin D1 and Caspase-3 protein in the liver.

Lane 1: HCT116 cell line, lane 2-5: control pig, lane 6-9: 50 mg/kg RS-treated pig. GAPDH served as the loading control. The original blots were cropped by Adobe photoshop. Full-length blots are presented in Supplementary Figure S2, S3 and S4.

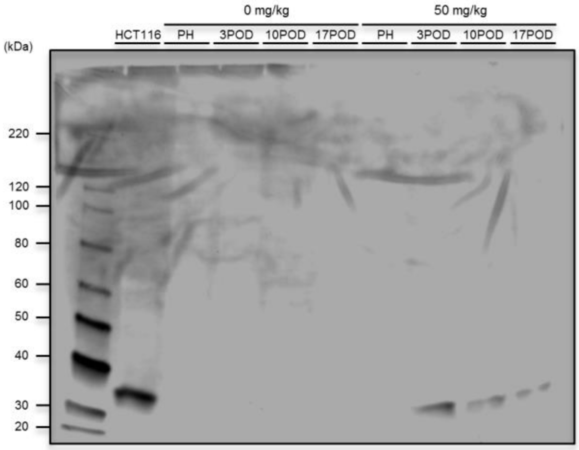

Supplementary Figure. S2. Western Blot analysis of Cyclin D1 protein. Western blotting demonstrated over-expression of cyclin D1 in RS-treated pigs after PH.

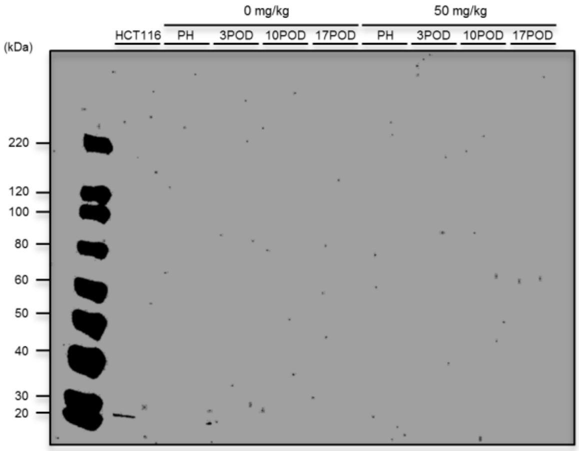

Supplementary Figure. S3. Western Blot analysis of Caspase-3 protein. The expression of this enzyme was not detected in control or RS-treated animals.

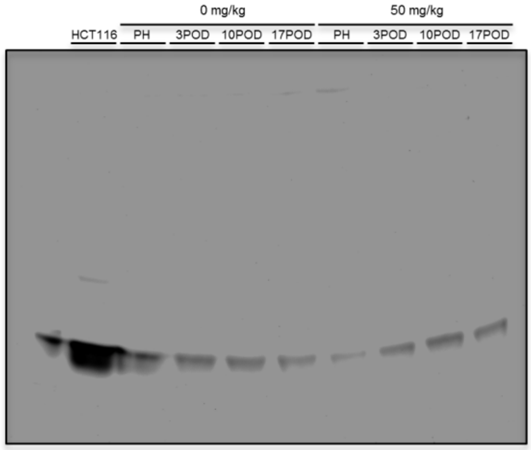

Supplementary Figure. S4. Western Blot analysis of GAPDH. The expression of GAPDH demonstrated the samples of control and treated animals have been loaded equally.
